# Modeling Parkinson’s disease in midbrain-like organoids

**DOI:** 10.1038/s41531-019-0078-4

**Published:** 2019-04-05

**Authors:** Lisa M. Smits, Lydia Reinhardt, Peter Reinhardt, Michael Glatza, Anna S. Monzel, Nancy Stanslowsky, Marcelo D. Rosato-Siri, Alessandra Zanon, Paul M. Antony, Jessica Bellmann, Sarah M. Nicklas, Kathrin Hemmer, Xiaobing Qing, Emanuel Berger, Norman Kalmbach, Marc Ehrlich, Silvia Bolognin, Andrew A. Hicks, Florian Wegner, Jared L. Sterneckert, Jens C. Schwamborn

**Affiliations:** 1 Luxembourg Centre for Systems Biomedicine (LCSB), Developmental and Cellular Biology, University of Luxembourg, Belvaux, Luxembourg; 2 DFG-Center for Regenerative Therapies, Technische Universität Dresden, Dresden, Germany; 3 Department of Cell and Developmental Biology, Max Planck Institute for Molecular Biomedicine, Münster, Germany; 4 Department of Neurology, Hannover Medical School, Hannover, Germany; 5 Institute for Biomedicine, Eurac Research, Affiliated Institute of the University of Lübeck, Bolzano, Italy; 6 Present Address: Neuroscience Discovery – Biology Department, AbbVie Deutschland GmbH & Co KG, Ludwigshafen, Germany

## Abstract

Modeling Parkinson’s disease (PD) using advanced experimental in vitro models is a powerful tool to study disease mechanisms and to elucidate unexplored aspects of this neurodegenerative disorder. Here, we demonstrate that three-dimensional (3D) differentiation of expandable midbrain floor plate neural progenitor cells (mfNPCs) leads to organoids that resemble key features of the human midbrain. These organoids are composed of midbrain dopaminergic neurons (mDANs), which produce and secrete dopamine. Midbrain-specific organoids derived from PD patients carrying the *LRRK2-*G2019S mutation recapitulate disease-relevant phenotypes. Automated high-content image analysis shows a decrease in the number and complexity of mDANs in *LRRK2-*G2019S compared to control organoids. The floor plate marker FOXA2, required for mDAN generation, increases in PD patient-derived midbrain organoids, suggesting a neurodevelopmental defect in mDANs expressing *LRRK2-*G2019S. Thus, we provide a robust method to reproducibly generate 3D human midbrain organoids containing mDANs to investigate PD-relevant patho-mechanisms.

## Introduction

The current in vitro disease modeling approaches are typically conducted with cultures of neurons grown under two-dimensional (2D) conditions. However, 2D cultures neglect physiologically relevant characteristics like the interaction between glia cells and neurons in a spatially organized microenvironment. Recently, a new class of 3D in vitro models has been developed to compensate for this deficit, the so-called organoids. The complex 3D structure of organoids consists of multiple region-specific cell types, which allow studying functional interactions^1–8^. Therefore, organoids represent a promising tool to model cell autonomous and non-cell autonomous aspects of neurodegenerative diseases on an organ-like level in contrast to individual neuronal subtypes in 2D. In this study, we used a chemically defined derivation of midbrain floor plate neural progenitor cells (mfNPCs), which can be efficiently differentiated into 2D midbrain dopaminergic neurons (mDANs) and 3D human midbrain-specific organoids (hMOs). To investigate Parkinson’s disease (PD)-relevant patho-mechanisms, we derived hMOs from PD patients carrying the *LRRK2*-G2019S mutation. We demonstrated that these organoids recapitulated key hallmarks of the pathology, such as reduced amounts of mDANs. Furthermore, we detected an increase of FOXA2 in patient-derived organoids compared to control organoids. This pinpoints at an impaired mDAN specification during hMO development. Thus, the generation of expandable mfNPCs provides an efficient and reproducible method to rapidly generate large quantities of functional mDANs suited for in vitro disease modeling.

## Results

### Generation of midbrain-specific organoids

Our objective was to generate 3D organoids that resembled the complexity of the human midbrain. Based on previous findings^9^, we first identified conditions that specifically promoted the formation of induced pluripotent stem cell (iPSC)-derived mfNPCs to generate a suitable starting population for the hMOs. The inhibition of bone morphogenetic protein (BMP) and tumor growth factor-β (TGFβ) signaling in combination with activation of SHH and WNT signaling enabled the generation of expandable neural stem cells (Supplementary Fig. 1a). Microarray profiling demonstrated that midbrain floor plate markers were highly expressed in these cells, in contrast to dorsal markers (IRX3, PAX3, PAX6), which were strongly downregulated (Supplementary Fig. 1b). Immunohistochemistry confirmed that these cells maintained the expression of midbrain floor plate markers EN1 and FOXA2 over several passages (Supplementary Fig. 1d). Differentiation efficiency of mfNPCs toward the dopaminergic lineage was determined by flow cytometry. After 14 days of mDAN differentiation, 59.94% (±10.6% SEM, mfNPC line P2-GC, *n* = 3) TH-positive cells were measured. Cells expressed multiple markers of mDAN fate and acquired essential functional electrophysiological properties during their differentiation (Supplementary Fig. 2). Therefore, mfNPCs represent an ideal starting population to generate hMOs.

**Fig. 1:**
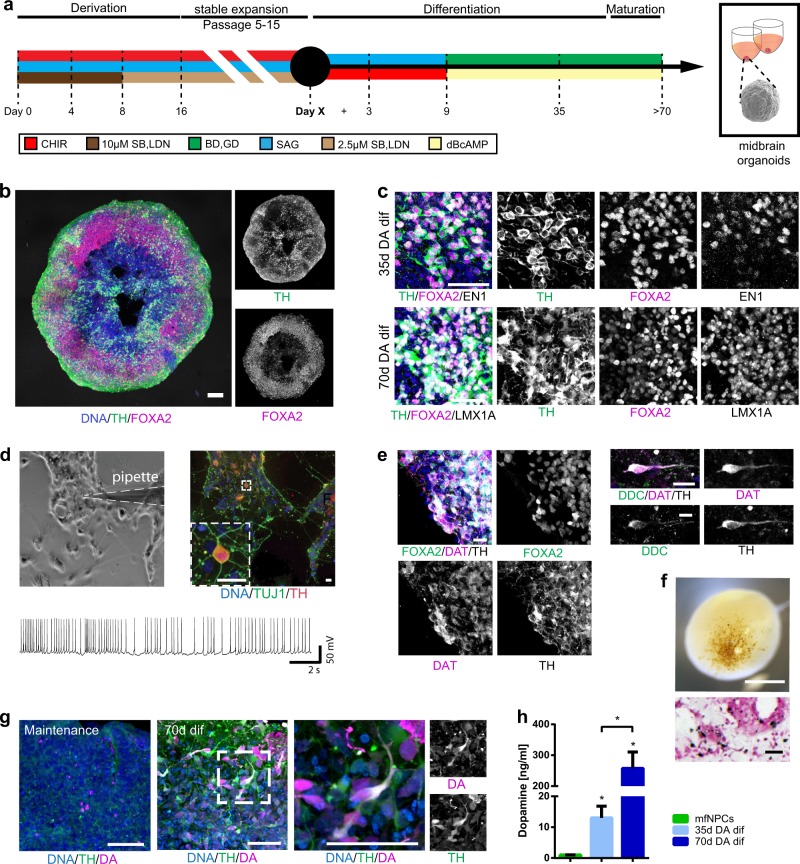
Generation of midbrain-specific organoids. **a** Illustration of the conditions used to differentiate spherical midbrain floor plate neural progenitor cells (mfNPCs) into human midbrain-specific organoids (hMOs). mfNPCs are stably expandable up to passage 15; day X indicates the start of dopaminergic differentiation. SB = SB-431542, LDN = LDN-193189, SAG = sonic hedgehog agonist, BD = brain-derived neurotrophic factor, GD = glial cell-derived neurotrophic factor. **b** Maximum intensity projection of midbrain dopaminergic neuron (mDAN) markers TH and FOXA2 in 70-day-old hMO sections. Scale bar is 100 μm. hMOs derived from mfNPC lines H1–3 (image shows line H3). **c** Immunohistological stainings of mDAN markers TH, FOXA2, EN1, and LMX1A in the center of hMO sections. Scale bar is 50 μm. hMOs derived from mfNPC lines H1–3 (images shows line H2). **d** Bright-field image showing neurons obtained from three-dimensional (3D) culture of line H4 where a recorded cell is attached to the patch-clamp pipette tip (×10 magnification). Scale bar is 20 µm. Representative current-clamp trace of the pace-making firing activity obtained from the recorded mDAN is shown. Experiment conducted with mfNPC lines H4, P2-GC, and P3-GC, in total 26 cells have been recorded, 12 of these showed a similar pattern; representative images and traces of line H4. **e** Representative immunohistological stainings of mDAN markers FOXA2, TH, dopamine decarboxylase (DDC), and dopamine transporter (DAT) at the edge of an organoid. Scale bar is 20 μm. hMOs were derived from mfNPC lines H1–4 (images show lines H3 and H1). **f** Appearance of dark granules in hMOs at day 255, derived from mfNPC line H1. Scale bar is 500 µm. Fontana-Masson staining reveals neuromelanin-like granules at the edge of the organoid section after 100 days in culture, derived from mfNPCs line H4. Scale bar is 50 µm. **g** Representative immunohistological staining of spherical maintenance mfNPCs and 70-day hMO sections (50 μm thickness, taken at the edge of a section) for TH and neurotransmitter dopamine (DA). Scale bar is 50 μm. hMOs derived from mfNPC lines H1–3 (images show line H3). **h** Quantitative analysis of DA extracted from the supernatant of mfNPCs and hMOs, *p < 0.05. The two time points analyzed in all the experiments were 35 and 70 days of differentiation. Data are presented as mean ± SEM (mfNPC line H3, 4 different passages, *n* = 4)

After initiating the neuronal patterning of mfNPCs, 3D cultures were kept under differentiation conditions and were analyzed after 35 and 70 days (Fig. 1 and Supplementary Fig. 3). High-content image analysis revealed that hMOs showed high efficiency to form mDANs (62.38% ± 3.5% SEM TH-positive cells after 35 days and 54.12% ± 5.8% SEM after 70 days of differentiation, mfNPC line H3, *n* = 7, calculated as described in Supplementary Table 3). The markers TH, FOXA2, EN1, and LMX1A were abundantly expressed (Fig. 1b, c and Supplementary Fig. 3b) and we could also identify additional ventral and midbrain-specific markers (Supplementary Fig. 3d). Additionally, pace making and evoked firing activity was recorded in TH-positive cells by whole-cell patch-clamp measurements (this was detected in 12 out of 26 neurons, Fig. 1d and Supplementary Fig. 3c). A mean frequency of 9 ± 2 Hz was detected, 81% of the cells displayed sodium currents and 100% potassium currents. Immunofluorescence analysis demonstrated that the dopaminergic nature of the recorded cell (Supplementary Fig. 3d). Besides TH, bona fide mDANs are further characterized by the expression of dopamine decarboxylase (DDC) and the dopamine transporter (DAT). We identified neurons that co-expressed TH/FOXA2/DAT as well as TH/DDC/DAT (Fig. 1e). Occasionally, we detected the formation of neuromelanin inclusions in older hMOs (>100 days of differentiation) (Fig. 1f). The presence of dopamine in hMOs was subsequently confirmed by immunostainings and enzyme-linked immunosorbent assay (ELISA) (Fig. 1g, h). This indicates that mDANs in organoids are functional and can produce and secrete dopamine.

### Disease modeling of PD patient-specific midbrain-specific organoids

**Fig. 2:**
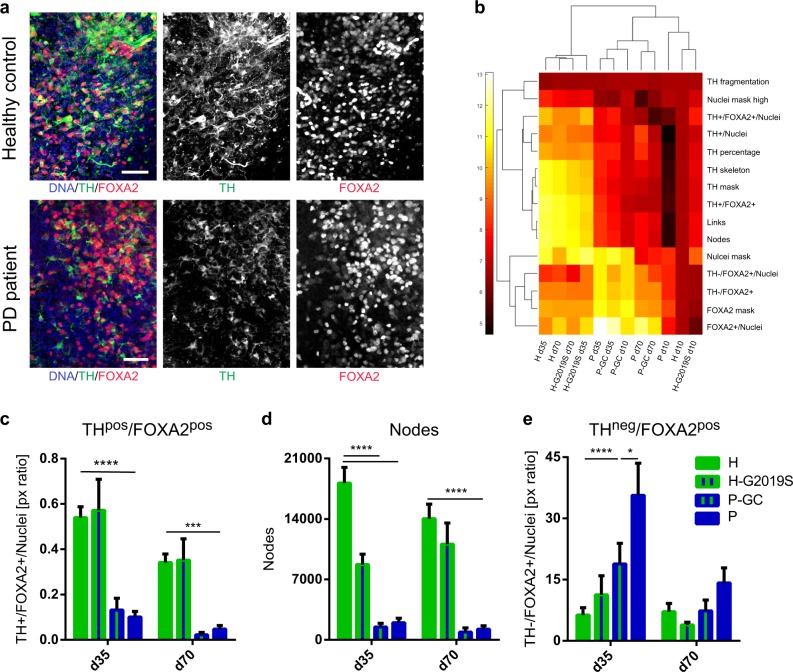
Disease modeling of Parkinson’s disease (PD) patient-derived midbrain-specific organoids. **a** Representative maximum intensity projection of TH-positive and FOXA2-positive cells in control and PD patient-specific human midbrain-specific organoids (hMOs) after 35 days of differentiation. Scale bar is 50 µm, magnification from the center of the organoid, images taken from line H3 and P3. **b** Heatmap comprising all features extracted by high-content automated image analysis. Dendrograms indicate clustering of genotypes and age (top) and features (left) and were obtained using the clustergram function in Matlab. **c**–**e** Quantification of **c** TH and FOXA2 double-positive signal, **d** number of nodes in the TH network, and **e** TH-negative, FOXA2-positive signal. Data are presented as mean ± SEM (and comprised of following numbers of hMO sections: healthy hMOs H (midbrain floor plate neural progenitor cell (mfNPC) line H3 and H4): *n* = 26 (d10), *n* = 32 (d35), *n* = 38 (d70); patient-derived hMOs P (mfNPC line P3 and P4): *n* = 23 (d10), *n* = 36 (d35), *n* = 31 (d70); isogenic control hMOs H-G2019S (mfNPC line H3-G2019S and H4-G2019S): *n* = 18 (d10), *n* = 29 (d35), *n* = 28 (d70); isogenic control hMOs P-GC (mfNPC line P3-GC and P4-GC): *n* = 21 (d10), *n* = 38 (d35), *n* = 22 (d70)). Relevant statistical significances determined by two-way analysis of variance (ANOVA), Tukey’s multiple comparisons test are indicated with asterisks: *p <0.05, ***p <0.001, ****p <0.0001 (complete statistical evaluation shown in Supplementary Table 4)

To assess the potential of mfNPC-derived hMOs for PD modeling, organoids were generated from healthy control (H-lines)- and from patient-specific iPSCs carrying the PD-associated *LRRK2-*G2019S mutation (P-lines). Their respective isogenic controls were also used in the study. The mutation was either introduced into the healthy control lines (H-G2019S lines) or corrected in the patient-specific lines (P-GC lines). mDANs were characterized after 10, 35, and 70 days of organoid differentiation with high-content image analysis (Fig. 2). At the beginning of the differentiation, hMOs from four different individuals (mfNPC line H3, H4, P2, and P3) as well as the corresponding isogenic controls (mfNPC line H3-G2019S, H4-G2019S, P2-GC, and P3-GC) developed similarly. Taking into consideration all analyzed features (see Supplementary Table 3), the heatmap shows that differences between the genotypes became evident when comparing hMOs at days 35 and 70 (Fig. 2b). For instance, healthy hMOs revealed a progressive increase in the TH percentage over time (d10 vs. d35, d10 vs. d70, H and H-G2019S), whereas patient-derived hMOs maintained lower levels throughout differentiation (d10 vs. d35, d10 vs. d70, P and P-GC). We detected a significant decrease of TH/FOXA2 double-positive signal in PD-derived compared to healthy control-derived hMOs after 35 days of differentiation (Fig. 2a). Introduction of the mutation into the healthy background or the correction of the mutation within the patient lines did not show the same effect (Fig. 2c). However, after evaluating the complexity of the dopaminergic neuronal network expressed as the number of nodes (dendrite bifurcation points) and links (number of branching)^10^, we indeed identified a significant reduction of this complexity in the patient-derived TH-positive neurons compared to control mDANs (Fig. 2d). This phenotype was reproduced with the introduction of the LRRK2-*G2019S* mutation in the healthy background. Strikingly, we observed a significant increase of TH-negative but FOXA2-positive progenitor cells in the patient-specific hMOs (Fig. 2a, e). The same outcome became apparent when comparing 35-day-old hMOs generated from P and P-GC lines. This suggests that the disease-associated decrease in mDANs in patient-specific hMOs might be caused by a neurodevelopmental defect, leading to an altered specification of mDANs.

## Discussion

Disease modeling and drug discovery in the field of PD require enormous amounts of disease-relevant cells like mDANs that can be produced in a rapid and reproducible way. Numerous published protocols describe the generation of ventral mDANs from human PSCs by replicating mDAN specification in vivo^11–14^. Although current protocols are based on the generation of LMX1A/FOXA2-positive midbrain floor plate progenitors, differentiations starting from PSCs are time consuming and typically result in cultures containing various neuronal identities^12,13,15,16^. Here, we report an approach to efficiently differentiate mDANs and hMOs by generating expandable mfNPCs. As neurons form functional networks with other neurons and non-neuronal cells in the brain, it is essential to expand our research of neurodegenerative diseases using 3D models that are able to reproduce these interactions. So far, no phenotypes for PD have been shown in any of the published human brain organoid models. Here, we provide a proof-of-principle study that hMOs harboring the *LRRK2*-G2019S mutation show PD-relevant phenotypes, including reduced number and complexity of mDANs, which also occur in PD patients’ brains^17,18^. Interestingly, we demonstrate a significant increase of FOXA2-positive progenitor cells in the patient-specific organoids. Since FOXA2 is required for the generation of mDANs, we hypothesize that this might be a compensatory response to an impaired specification of mDANs promoted by the mutated *LRRK2* gene. Similar compensatory mechanisms have been described in PD before^19^ and might represent an attempt to counteract neurodevelopmental defects induced by PD-specific mutations. While introducing also isogenic control hMOs in this study, we could confirm that the introduction of the *LRRK2*-G2019S mutation caused deleterious effects on the complexity of mDANs within a healthy background. On the contrary, *LRRK2*-G2019S gene correction within a PD patient background is not sufficient to rescue this effect. This supports the hypothesis that the genetic background of PD patients can influence the degeneration of mDANs^10^. These findings show that mfNPCs and thereof derived mDANs as well as 3D hMOs represent powerful new tools for in vitro disease modeling. The patient-specific nature of these models also opens promising avenues for future personalized medicine approaches.

## Methods

### Pluripotent stem cell culture and smNPC culture

The iPSCs used to derive neural progenitor cells were generated for and described in the study of Reinhardt et al.^9^ and Qing et al.^20^ and listed in the Supplementary Table 1. The cells were tested with the LookOut Mycoplasma PCR Detection Kit (Sigma-Aldrich) to exclude mycoplasma contamination. The cell line authentication via Sanger cell sequencing was performed by Microsynth Seqlab in Göttingen, Germany.

The generation of small molecule neural precursor cells (smNPCs) is described in detail in the study of Reinhardt et al.^9^

### Derivation of mfNPCs

For generation of mfNPCs from pluripotent stem cells, iPSCs were split as single cells and plated on feeder layers. Three to four days after splitting, colonies were detached using 2 mg/ml collagenase IV. Pieces of colonies were collected by sedimentation and resuspended in N2B27 medium supplemented with 10 µM SB-431542 (SB, Abcam), and 150 nM LDN-193189 (LDN, Millipore) for neural induction, as well as 5 µM ROCK inhibitor, 3 µM CHIR99021 (CHIR, Tocris), 200 µM ascorbic acid (AA, Sigma), and 0.5 µM SAG (Cayman). Cells were cultured in Petri dishes to prevent attachment. N2B27 medium consists of Dulbecco’s modified Eagle’s medium/Nutrient Mixture F12 (DMEM-F12) (Invitrogen)/Neurobasal (Invitrogen) 50:50 with 1:200 N2 supplement (Invitrogen), and 1:100 B27 supplement lacking vitamin A (Invitrogen) with 1% penicillin/streptomycin/glutamine (Biochrome). The medium was exchanged on days 2 and 4 with N2B27 medium supplemented with the same small molecule supplements, but without ROCK inhibitor. On day 6, embryoid bodies (EBs) showing neuroepithelial outgrowth without visible mesodermal/endodermal differentiation were collected and triturated with a 1000 µl pipette into smaller pieces. Then, they were plated on Matrigel-coated 12-well plates at a density of about 15–20 EBs per well in N2B27 medium supplemented with 10 µM SB, 150 nM LDN, 200 µM AA, 3 µM CHIR, and 0.5 µM SAG. Two days after plating the EBs, the medium was changed to mfNPC expansion medium (N2B27 medium with 2.5 µM SB, 100 nM LDN, 3 µM CHIR, 200 µM AA, and 0.5 µM SAG). The first split was performed 2–4 days after plating. During the early passages, it is important to keep the cells at high densities to increase survival. For the first split seed 500,000 cells per 12-well in mfNPC expansion medium containing an additional 5 µM ROCK inhibitor. In the next two passages, ~350,000 cells per 12 well are seeded to keep high densities and increase cell survival. All remaining splits are performed at a 1:5 to 1:10 ratio. After five passages, cultures are almost free of contaminating non-neuronal progenitor cells.

To derive mfNPCs under 3D conditions, iPSCs were detached using Accutase (Sigma) and collected in EB medium, consisting of knockout DMEM (Invitrogen) with 20% knockout serum replacement (Invitrogen), 100 µM β-mercaptoethanol (Gibco), 1% nonessential amino acids (Invitrogen), 1% penicillin/streptomycin/glutamine (Invitrogen), freshly supplemented with 10 µM SB-431542 (SB, Ascent Scientific), 250 nM LDN-193189 (LDN, Sigma), 3 µM CHIR99021 (CHIR, Axon Medchem), 0.5 µM SAG (Merck), and 5 µM ROCK inhibitor (Sigma). EBs were formed with 2,000 iPS cells each, using AggreWell 400 (Stemcell Technologies). EBs were harvested in EB medium without ROCK inhibitor after 24 h and transferred to a non-treated tissue culture plate (Corning). On day 2, the medium was replaced with N2B27 (as described above) supplemented with 10 µM SB, 250 nM LDN, 3 µM CHIR, and 0.5 µM SAG. The medium was exchanged with the same medium including 200 µM AA (Sigma) on days 4 and 6. On day 8, EBs with neuroepithelial outgrowth were collected, triturated with a 1000 µl pipette into smaller pieces, and transferred in a 1:10 ratio to an ultra-low attachment 24-well plate (Corning). For the following passages, cells were split with 1× TrypLE Select Enzyme (Gibco)/0.5 mM EDTA (Invitrogen) in 1× phosphate-buffered saline (PBS) and 10,000 to 20,000 cells per 96-well ultra-low attachment plate (round bottom, Corning) were seeded. The cells were always kept under 3D culture conditions and from passage 1 on cultured in N2B27 medium freshly supplemented with 2.5 µM SB, 100 nM LDN, 3 µM CHIR, 200 µM AA, and 0.5 µM SAG. After four to five passages, mfNPCs were used as a starting population for midbrain-specific organoids.

### Expansion of mfNPCs

mfNPCs were cultured on Matrigel-coated 12-well cell-culture plates (Greiner). mfNPC expansion medium consisted of N2B27 freshly supplemented with SB, LDN, CHIR, SAG, and AA, with a medium change every 2 days. Typically, cells were split every 5 or 6 days at a ratio of 1:10 up to 1:15. For splitting, cells were digested into single cells for about 5 to 10 min at 37 °C with prewarmed Accutase (Sigma). Cells were diluted in DMEM (Biochrome) with 0.1% bovine serum albumin (BSA) fraction V (Invitrogen) for centrifugation at 200 × *g* for 5 min. The cell pellet was resuspended in fresh mfNPC expansion medium and plated on Matrigel-coated cell culture dishes. For coating, Matrigel was diluted to a final dilution of 1:100 in knockout DMEM (Invitrogen) prior to coating 500 µl per well of a 12-well plate overnight. Coated plates were wrapped with parafilm and kept in the fridge for up to one month.

To perform splitting of the 3D cultured mfNPCs, spherical colonies were collected and treated with 1× TrypLE Select Enzyme(Gibco)/0.5 mM EDTA (Invitrogen) in 1× PBS for about 5 to 10 min at 37 °C, followed by gentle pipetting to generate single cells. After re-seeding 10,000 to 20,000 cells per well of an ultra-low attachment, 96-well round bottom plate (Corning) was centrifuged for 3 min at 200 × *g* to assure the aggregation of single cells at the bottom of the well. Additionally, 5 µM ROCK inhibitor was added to the medium after passaging the cells. The cells were split every 7 to 14 days and the medium was changed every third day. mfNPCs were usually expanded from passages 5 to 15.

### Differentiation of mfNPCs

For the differentiation into mDANs, 350,000 mfNPCs were seeded into a 12-well. Since SB and LDN are detrimental for differentiation, both small molecules were left out when seeding the cells. mfNPC expansion medium (without SB and LDN) was changed 2 days after seeding to N2B27 medium with 0.5 µM SAG, 0.7 µM CHIR, and 200 µM AA. After 4 days of patterning, maturation of the neurons was induced by changing the medium to N2B27 medium with 10 ng/ml brain-derived neurotrophic factor (BDNF, Peprotech), 10 ng/ml glial cell-derived neurotrophic factor (GDNF, Peprotech), 200 µM AA, 500 µM dibutyryl camp (Sigma), 1 ng/ml TGF-β3 (Peprotech), and 2.5 ng/ml ActivinA (Peprotech). When cultures became over-confluent during maturation, they were split into single cells using Accutase, or as small clumps using a cell scraper. To increase the maturation of differentiating cultures, the maturation medium was supplemented with 5–10 µM dual antiplatelet therapy (DAPT) (Cayman). Cultures were analyzed after 8–10 days under maturation conditions, unless otherwise indicated.

To generate midbrain-specific organoids, 3,000 cells were seeded per well to an ultra-low attachment 96-well round bottom plate and kept under maintenance conditions for 7 days. To start the pre-patterning, LDN and SB were withdrawn and after 3 additional days, the concentration of CHIR was reduced to 0.7 µM similar to 2D culturing. On day 9 of differentiation, the medium was changed to neuronal maturation medium including 10 µM DAPT, as described above. The organoids were kept under static culture conditions with media changes every third day for 10, 35, or 70 days.

### Immunofluorescence staining

Cells were fixed for 20 min with 4% paraformaldehyde (PFA, Electron Microscopy Sciences) in PBS and washed twice with PBS (Lonza). Permeabilization and blocking were performed simultaneously using 0.1% Triton X-100 (Sigma), 1% BSA (Sigma), and 10% fetal calf serum in PBS for 45 min. The cells were washed with 0.1% BSA in PBS, and the primary antibodies were applied overnight at 4 °C in 0.1% BSA in PBS. The next day, the cells were washed with 0.1% BSA in PBS and incubated with secondary antibodies for 1 h at room temperature (RT). Finally, cells were washed three times with 0.1% BSA in PBS-T (0.005% Tween-20), including a Hoechst 33342 counterstaining for nuclei in the second washing step. Cells were mounted using Vectashield (Vector Labs) and imaged on a Zeiss PALM/Axiovert fluorescence microscope. When necessary, images were merged using ImageJ and Adobe Photoshop.

The primary antibodies used in this study are summarized in Supplementary Table 2. All secondary antibodies were obtained from Invitrogen and were conjugated to Alexa Fluor fluorochromes. Immunofluorescence staining for characterizing 2D-derived mfNPCs was performed with the cell lines mfNPC H1, P1-GC, and P2-GC (see Supplementary Table 1).

hMOs were fixed with 4% PFA overnight at 4 °C and washed three times with PBS for 15 min. After treatment, they were embedded in 3–4% low-melting point agarose in PBS. The solid agarose block was sectioned with a vibratome (Leica VT1000s) into 50 µm sections. The sections were blocked on a shaker with 0.5% Triton X-100, 0.1% sodium azide, 0.1% sodium citrate, 2% BSA, and 5% normal goat or donkey serum in PBS for 90 min at RT. Primary antibodies were diluted in the same solution but with only 0.1% Triton X-100 and were applied for 48 h at 4 °C.

After incubation with the primary antibodies (s. Supplementary Table 2), sections were washed three times with PBS and subsequently blocked for 30 min at RT on a shaker. Then, sections were incubated with the secondary antibodies in 0.05% Tween-20 in PBS for 2 h at RT and washed with 0.05% Tween-20 in PBS and Milli-Q water before they were mounted in Fluoromount-G mounting medium (Southern Biotech).

STAINperfect Immunostaining Kit (ImmuSmol) was used according to the manufacturer’s protocol to detect dopamine. Sections were co-stained with chicken anti-TH primary antibody (Abcam), and nuclei were counterstained with Hoechst 33342 (Invitrogen).

For qualitative analyses, three randomly selected fields per organoid section were acquired with a confocal laser scanning microscope (Zeiss LSM 710) and images were further processed with the OMERO Software. For these immunofluorescence stainings of 3D derived mfNPCs and hMOs, the cell lines mfNPC H1, H2, H3, and H4 were used (see Supplementary Table 1).

### Flow cytometry

Flow cytometry analysis was performed to quantify the differentiation efficiency of 2D mDAN cultures. To do so, we analyzed three independently differentiated wells of mfNPC P2-GC of three consecutive passages (*n* = 3) after 14 days of differentiation. In order to generate a single-cell suspension, differentiated cultures were washed once with PBS and subsequently dissociated at 37 °C using Accutase. Cells were further dissociated by pipetting gently up and down as well as by filtering them carefully through a 0.75 µm cell strainer. After centrifugation at 200 × *g* for 5 min, the cell pellet was resuspended in 2 ml PBS and the same volume of 8% PFA in PBS was added dropwise to fix the cells for 10 min at RT. Subsequently, the same volume of 0.1% BSA in PBS was added to the suspension. To remove the PFA, the cell suspension was centrifuged and washed once with 0.1% BSA in PBS. The pellet was resuspended in in 0.1% BSA in PBS and the suspension transferred into a 1.5 ml tube. After centrifugation, the cell pellet was resuspended in permeabilization/blocking solution (see section Immunofluorescence staining) and incubated for 20 min at RT. Permeabilization/blocking solution was withdrawn by centrifugation and the resulting cell pellet was resuspended in primary antibody solution. The incubation with the primary antibody rabbit anti-TH (1:300, Santa Cruz) was performed under shaking conditions overnight at 4 °C. On the following day, cultures were washed once with 0.1% BSA in PBS, and incubated with the secondary antibody for 1 h at RT. All secondary antibodies were obtained from Invitrogen and were conjugated to Alexa Fluor fluorochromes. Finally, cells were washed twice with 0.1% BSA in PBS-T (0.005% Tween-20), including a Hoechst 33342 counterstaining for nuclei in the second washing step. After centrifugation, the pellet was resuspended in FACS buffer (0.1% BSA in PBS). To set the gates appropriately, we stained for each marker individually and included samples that have been stained with Hoechst individually. Flow cytometry was performed using BD LSRFortessa Cell Analyzer.

### Image analysis

Immunofluorescence 3D images of hMOs were analyzed in Matlab (Version 2017b, Mathworks). The in-house developed image analysis algorithms automate the segmentation of nuclei and neurons, with structure-specific feature extraction (see Supplementary Table 3).

The image preprocessing for the segmentation of nuclei was computed by convolving the raw Hoechst channel with a Gaussian filter. By selecting a pixel threshold to identify apoptotic cells, a pyknotic nuclei mask was identified and subtracted from the nuclei mask.

For the segmentation of dopaminergic neurons, a median filter was applied to the raw TH channel to generate TH mask. A skeleton of the TH mask was generated with a thinning function and was used to identify nodes and links as total number of bifurcation points and total number of linking elements, respectively. For SOX2, CC3, and FOXA2 masks, a median filter was applied followed by a bwareaopen to remove all connected components smaller than 100. SOX2 and FOXA2 masks were identified within the nuclei masks of each image. The expression levels of the mentioned markers were expressed in two ways: (i) positive pixel of the marker, normalized by the pixel count of Hoechst; (ii) cells positive for a marker expressed as a percentage of the total number of cells. In this latter case, the nuclei were segmented and a watershed function was applied. Considering the high cell density of the specimens, steps to ensure high quality in the segmentation process were implemented and nuclei with a size higher than 10,000 pixels were removed. In the nuclei successfully segmented as a single element, a perinuclear zone was identified. In case the marker or interest was positive in at least 1% of the perinuclear area, that cell was considered positive.

For quantitative analyses of SOX2 and CC3 marker expression in hMOs, mfNPC lines H1, H2, and H3 were used. With a confocal laser scanning microscope (Zeiss LSM 710), three randomly selected fields per organoid section were acquired of three different organoid derivations. In total 27 images per time point were analyzed with above-described image analysis algorithms. To evaluate the TH signal, 21 field of hMOs derived of seven consecutive passages of mfNPC H3 were acquired.

For the comparison of PD patient-derived and healthy hMOs, as well as their isogenic controls, cell lines mfNPC H3, H3-G2019S, H4, H4-G2019S, P3, P3-GC, P4, and P4-GC were used (see Supplementary Table 1). hMOs were generated minimum five times per line from consecutive 3D mfNPC cultures. The entire organoid sections of 50 µm thickness were acquired with an Operetta High-Content Imaging System (Perkin -Elmer) and analyzed with above-described image analysis algorithms.

### Dopamine ELISA

Dopamine Research ELISA (LDN) was performed for the quantitative determination of dopamine secreted by hMO cell line H3 (see Supplementary Table 1). Supernatant of 12 hMOs per condition was pooled, 200 µl was diluted 1:10 with a HCl buffer (0.01 N HCl, 4 mM Na_2_O_5_S_2_, and 1 mM EDTA), and snap frozen in liquid nitrogen. Samples were selected from four independently generated hMO batches, and differentiated from consecutive 3D mfNPC cultures. The ELISA was performed according to the manufacturer’s instructions with 10 µl sample volume.

### Fontana–Masson staining

hMOs of line mfNPC H4 were fixed at day 100 in 4% PFA overnight at 4 °C on a shaker, washed three times in PBS, and dehydrated in 30% sucrose overnight until the organoid was entirely equilibrated with the sucrose solution. Next, cryosectioning was performed using a Cryostat (Leica) to a thickness of 10 µm. The sections were dried overnight and stained with a Fontana–Masson Staining Kit (Abcam) according to the manufacturer’s protocol. The sections were mounted in Entellan® rapid mounting medium (Merck) and images were acquired using a stereomicroscope (Nikon SMZ25).

### Quantitative RT-PCR

For total RNA extraction of 3D cultured cells, typically 8 spherical mfNPC colonies and 12 hMOs per line (mfNPC line H1–4, see Supplementary Table 1) were collected and snap frozen in liquid nitrogen. Afterwards, they were lysed with 1 ml QIAzol lysis reagent (Qiagen), homogenized first with a needle, and then with QIAshredder columns (Qiagen). According to the manufacturer’s instructions, RNeasy Mini Kit (Qiagen) as well as DNase I Amplification Grade (Sigma-Aldrich) was used to isolate the RNA. After conducting reverse transcription by following the protocol of the High Capacity RNA to DNA Kit (Thermo Fisher Scientific), qRT-PCRs were performed using TaqMan Gene Expression Master Mix (Thermo Scientific), and the following Taqman® probes: RPL37A (Hs00902901_m1), FOXA2 (Hs00232764_m1), LMX1A (Hs00892663_m1), EN1 (Hs00154977_m1), and TH (Hs00165941_m1). Amplification of 5 ng of original RNA was performed in a LightCycler R480 (Roche) as follows: an initial denaturing step, 10 min at 95 °C, 40 cycles of denaturation for 15 s at 95 °C, annealing for 30 s at 60 °C, and elongation for 30 s at 72 °C. The expression levels were normalized relative to the expression of the housekeeping gene *RPL37A* using the comparative Ct method 2−ΔΔCt. Expression patterns of hMOs were compared to the expression levels of mfNPCs cultured under 3D conditions, which were set to 1.

### Whole-genome expression analysis

Microarray analysis was performed as described previously^21^. Briefly, 500 ng RNA of smNPCs and mfNPCs (cell lines P1-GC and P2-GC) were processed using a linear amplification kit (Ambion), quality controlled on a 2100 Bioanalyzer (Agilent), and hybridized on Illumina human-12 V3 expression BeadChips. Background subtraction, normalization, and differential expression analysis were performed using BeadStudio. Here smNPCs are used as a well-described^9^ neural progenitor cell line to which the mfNPCs are compared.

### Patch-clamp electrophysiology

mfNPC lines P1-GC and P2-GC were plated on Matrigel-coated 35-mm culture dishes (Greiner) and differentiated as 2D cultures described above for 2 or 4 weeks. Whole-cell patch-clamp recordings of neurons were performed at 20–22 °C under optical control (Zeiss inverted microscope) as reported previously^22^. The internal pipette solution consisted of 153 mM KCl, 1 mM MgCl_2_, 10 mM HEPES, 5 mM EGTA, and 2 mM Mg-ATP, adjusted to pH 7.3 with KOH (305 mOsm). The external bath solution contained 142 mM NaCl, 8 mM KCl, 1 mM CaCl_2_, 6 mM MgCl_2_, 10 mM glucose, and 10 mM HEPES, adjusted to pH 7.4 with NaOH (325 mOsm). Neuronal recordings were low-pass filtered at 1–5 kHz, digitized at 10 kHz using an EPC-10 amplifier (HEKA), and analyzed with Patch Master (HEKA).

hMO lines H4, P2-GC, and P3-GC were dissociated after 60–65 days in culture using the NeuroCult™ Enzymatic Dissociation Kit for Adult CNS Tissue (Stemcell Technologies) according to the manufacturer’s protocol and patch-clamp recordings (*n* = 26) were carried out 5–15 days after dissociation. Whole-cell recordings in voltage- and current-clamp modes were performed in a temperature-controlled recording chamber (35–37 °C) mounted on an inverted Eclipse-Ti microscope (Nikon, Tokyo, Japan) and using a MultiClamp 700B amplifier (Molecular devices, LLC). Voltage- and current-command protocols and data acquisition were performed using the pClamp 10.0 software and the Digidata 1550 interface (Molecular Devices, LLC). Data were low-pass filtered at 3 kHz and sampled at 10 kHz. Patch electrodes, fabricated from thick borosilicate glass capillaries, were made using a Sutter P-1000 puller (Sutter Instruments) to a final resistance of 4–6 MΩ when filled with the intracellular solution containing (in mM): 120 K-gluconate, 25 KCl, 10 EGTA, 10 HEPES, 1 CaCl_2_, 4 Mg-ATP, 2 Na-GTP, and 4 Na2-phosphocreatine (pH 7.4, adjusted with KOH). Cells were perfused with a Krebs solution containing (in mM): 129 NaCl, 5 KCl, 2 CaCl_2_, 1 MgCl_2_, 30 d-glucose, 25 HEPES, pH 7.3, with NaOH. Voltage-clamp recordings (Vh = −60 mV) of evoked ionic currents were performed by applying a voltage step protocol (from −60 to +60 mV, 300 ms of duration). Spontaneous action potentials were recorded in a gap-free mode, while the evoked firing activity was evaluated by applying long steps at different current intensities (50 pA increments). Series resistance was monitored during the experiments and recordings with changes over 20% of its starting value were discarded.

### Statistical analyses

2D experiments were performed with three different cell lines (mfNPC H1, P1-GC, and P2-GC), which have been published previously^9^. All hMO immunostainings and quantitative reverse transcription PCR experiments were performed with three independently generated organoid cultures from three to four different cell lines H1–4 (*n* = 9–12, see Supplementary Table 1). The dopamine ELISA was conducted with four different passages of mfNPC line H3 (*n* = 4). Gaussian distribution was evaluated by performing D’Agostino and Pearson omnibus normality test. According to this distribution, either a Grubbs’ test to detect significant outliers and an unpaired *t* test with Welch’s correction was carried out or a nonparametric Kolmogorov–Smirnov test was performed to evaluate statistical significance.

The image analysis for PD phenotyping was conducted with several passages of PD patient-derived and healthy hMOs, as well as their isogenic controls (mfNPC line H3, H3-G2019S, H4, H4-G2019S, P3, P3-GC, P4, and P4-GC, see Supplementary Table 1). A two-way analysis of variance and Tukey’s multiple comparisons test was performed to evaluate statistical significance (see Supplementary Table 4). Data are presented as mean ± SEM.

All analyses were performed with multiple cell lines; in the figures always a representative image looking similar to the images obtained from all used lines is displayed.

### Ethics statement

Informed consent was obtained from all individuals donating samples to this study prior to the donation using a written form and protocol previously approved by the institutional review board: Ethik-Kommission der Medizinischen Fakultät am Universitätsklinikum Tübingen. In vitro experiments were carried out with existing cell lines obtained from previous studies. Cell lines used in this study are summarized in Supplementary Table 1.

### Reporting Summary

Further information on research design is available in the Nature Research Reporting Summary linked to this article.

## Supplementary information

Supplementary Information

Life Sci Reporting Summary

## Data Availability

The data that support the findings of this study are publically available at this 10.17881/lcsb.20192701.01. The microarray data that support the findings of this study have been deposited in Gene Expression Omnibus (GEO) under the accession code GSE127967.

## Supplementary information

**Supplementary information** accompanies the paper on the *npj Parkinson’s Disease* website (10.1038/s41531-019-0078-4).

## Code availability

The codes used for image analysis in this study are available via github: https://github.com/LCSB-DVB.
