# Supplementary material for: Modeling Parkinson’s disease in midbrain-like organoids
Source: NPJ Parkinsons Dis. 2019 Apr 5;5:5. doi: 10.1038/s41531-019-0078-4 (PMC6450999; doi:10.1038/s41531-019-0078-4)
Supplement: Supplementary file 2 — Life Sci Reporting Summary [file 41531_2019_78_MOESM2_ESM.pdf]

Supplementary Material

Modeling Parkinson's disease in midbrain-like organoids

Lisa M. Smits[1,§], Lydia Reinhardt[2,3,§], Peter Reinhardt[2-4], Michael Glatza[2,3], Anna S. Monzel[1], Nancy Stanslowsky[5], Marcelo D. Rosato-Siri[6], Alessandra Zanon[6], Paul M Antony[1], Jessica Bellmann[2], Sarah M. Nicklas[1], Kathrin Hemmer[1], Xiaobing Qing[1], Emanuel Berger[1], Norman Kalmbach[5], Marc Ehrlich[3], Silvia Bolognin[1], Andrew A. Hicks[6], Florian Wegner[5], Jared L. Sterneckert[2,3*] and Jens C. Schwamborn[1*]

[1]Luxembourg Centre for Systems Biomedicine (LCSB), Developmental and Cellular Biology, University of Luxembourg, Belvaux Luxembourg

[2]DFG-Center for Regenerative Therapies, Technische Universität Dresden, Dresden, Germany

[3]Department of Cell and Developmental Biology, Max Planck Institute for Molecular Biomedicine, Münster, Germany

[4]Currently located at AbbVie Deutschland GmbH & Co KG, Neuroscience Discovery– Biology Department, Ludwigshafen, Germany

[5]Department of Neurology, Hannover Medical School, Hannover, Germany

[6]Institute for Biomedicine, Eurac Research, Affiliated Institute of the University of Lübeck, Bolzano, Italy

[§]These authors contributed equally and thus share first authorship.

[*]Corresponding authors

Prof. Dr. Jens C. Schwamborn
jens.schwamborn@uni.lu
Dr. Jared L. Sterneckert
Jared.Sterneckert@tu-dresden.de

Supplementary Figures and Tables

Supplementary Figure 1 Derivation of mfNPCs.

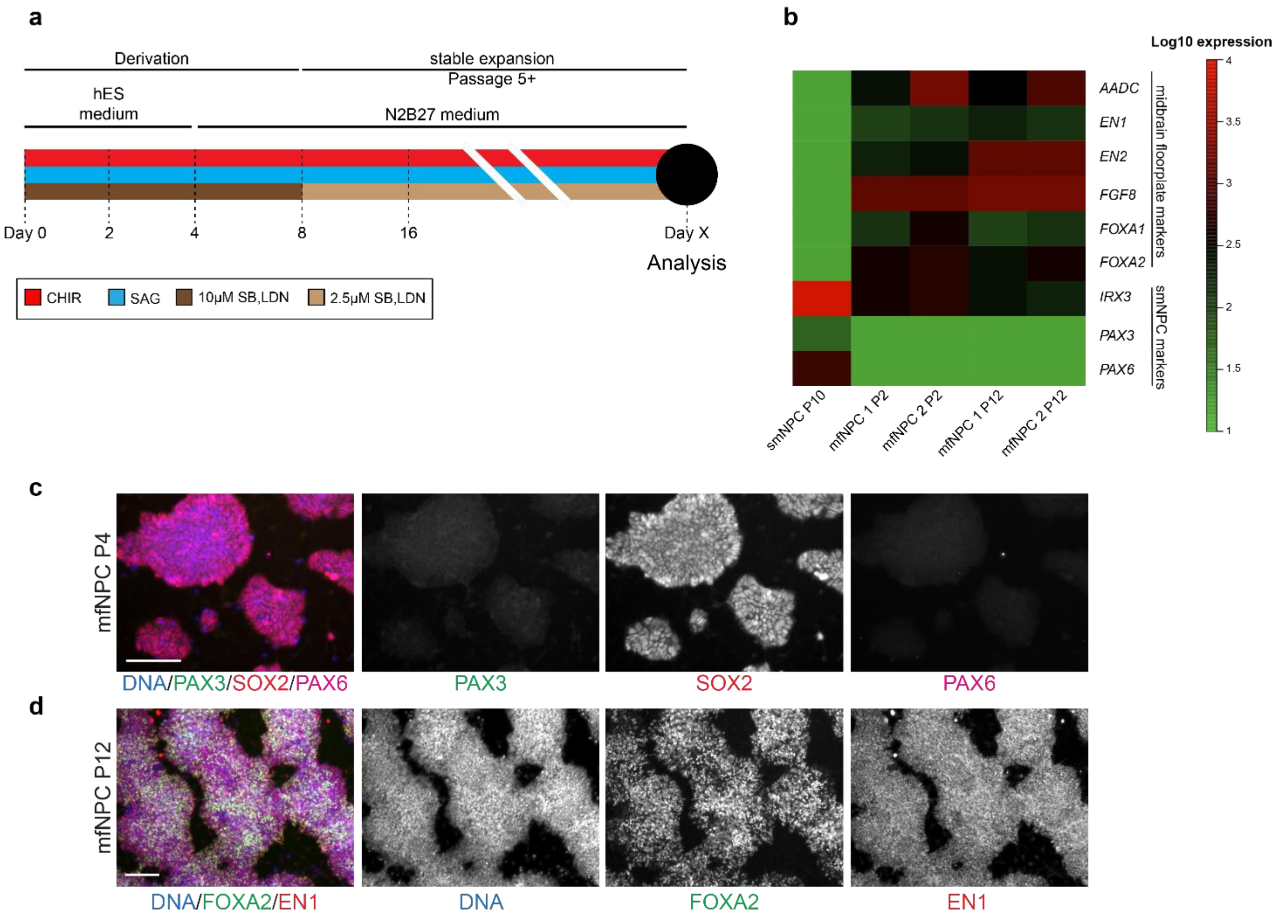

Supplementary Figure 1: Derivation of mfNPCs. (a) Schematic illustration of mfNPC derivation and expansion. SB = SB-431542, LDN = LDN-193189, SAG = sonic hedgehog agonist. (b) Microarray profiling displayed as a heatmap for selected markers of regional cell fate identity in small molecule neural precursor cells (smNPCs) and midbrain floor plate neural precursor cells (mfNPCs). (c) Immunostaining shows that mfNPCs lack PAX3 and PAX6, which are not expressed in the midbrain floor plate. Scale bar is 100 μm. (d) Immunofluorescence confirms that mfNPCs co-express FOXA2 and EN1, which are markers of the midbrain floor plate. Scale bar is 100 μm. Experiments were conducted with mfNPC lines H1, P1-GC, P2-GC, here representative images are shown for line P1-GC, which looked similar to the other cell lines.

Supplementary Figure 2 Differentiation potential of mfNPCs for mDANs.

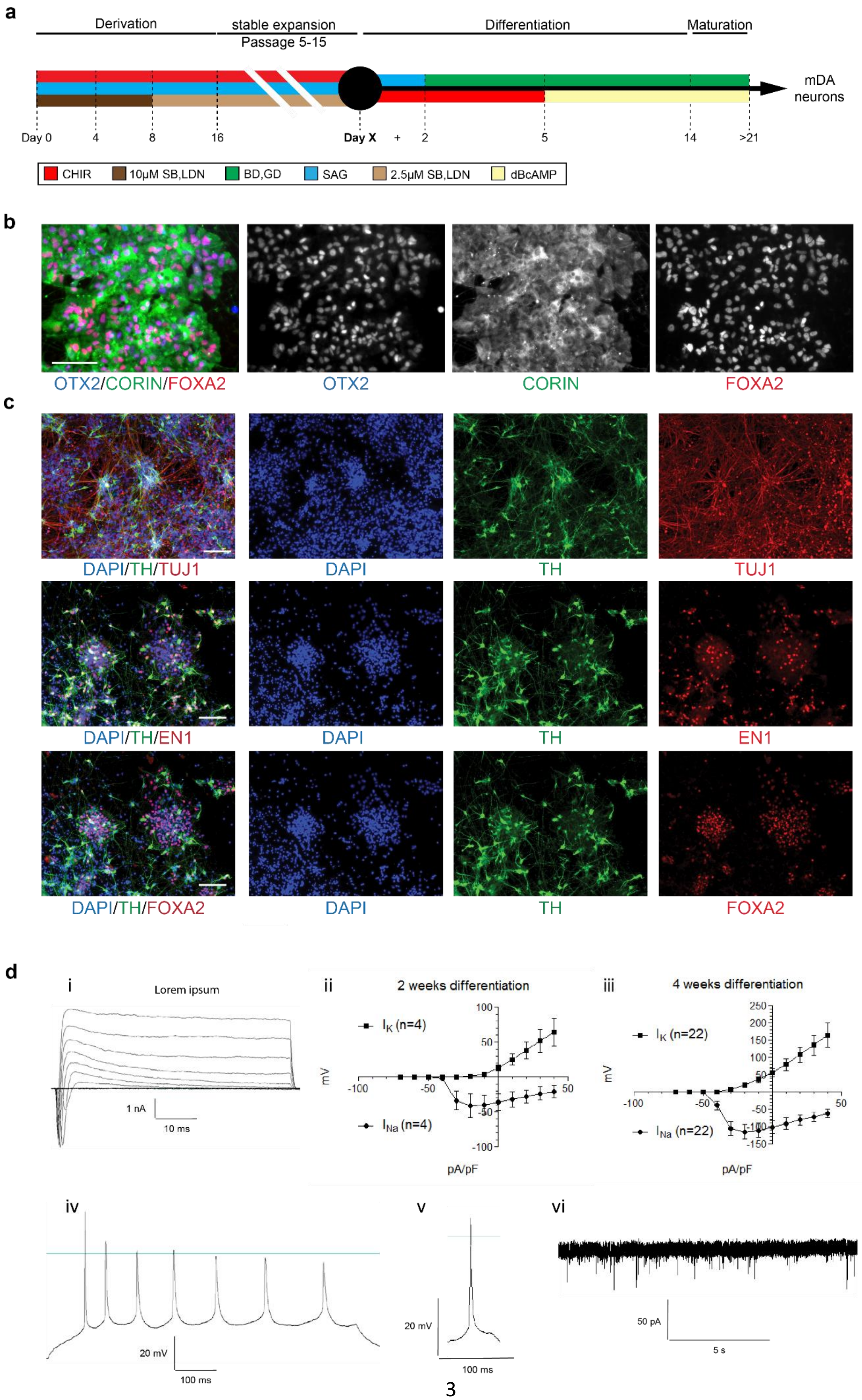

Supplementary Figure 2: Differentiation potential of mfNPCs for mDANs. Where indicated, analyses were performed with multiple cell lines (details see below) and a representative image looking similar to the images obtained from all used lines, is displayed. (a) Schematic illustration of the conditions used to differentiate mfNPCs into mDANs under 2D conditions. BD = BDNF, GD = GDNF. (b) mfNPCs were differentiated for four days and immunostained for CORIN and OTX2, which are markers of mDAN progenitors. (c) Immunostaining for the midbrain dopaminergic markers EN1, FOXA2, and TH after 14 days of differentiation. Scale bars are 100 µM. Stainings were performed with mfNPC lines H1, P1-GC, P2-GC, here representative images are shown for line P1-GC. (d) Patch-clamp recordings of voltage-gated ion currents, action potentials and synaptic activity of mfNPC-derived neurons after four weeks of differentiation indicate the development of essential functional properties during differentiation. (i) Representative voltage-gated sodium inward and potassium outward currents of a mfNPC-derived neuron recorded in whole-cell voltage-clamp mode by stepwise depolarizations in 10 mV increments from a holding potential of -70 to 40 mV. (ii-iii) Ion currents normalized for cell sizes based on the capacitance of the cell membrane (pA/pF) after differentiation for two weeks (ii) and four weeks (iii). Data are presented as means ± SEM. (iv) Example of neuron firing repetitive action potentials upon depolarization in the current-clamp mode. (v) Some neurons were also able to spike single action potentials spontaneously (same cell as in i after four weeks of differentiation). (vi) Spontaneous synaptic activity of neurons with miniature postsynaptic currents recorded in voltage-clamp mode. Recordings were performed with neurons derived from mfNPC lines P1-GC and P2-GC, four cells were recorded after two weeks of differentiation, 22 cells were recorded after four weeks of differentiation.

Supplementary Figure 3 Characterization of midbrain-specific organoids.

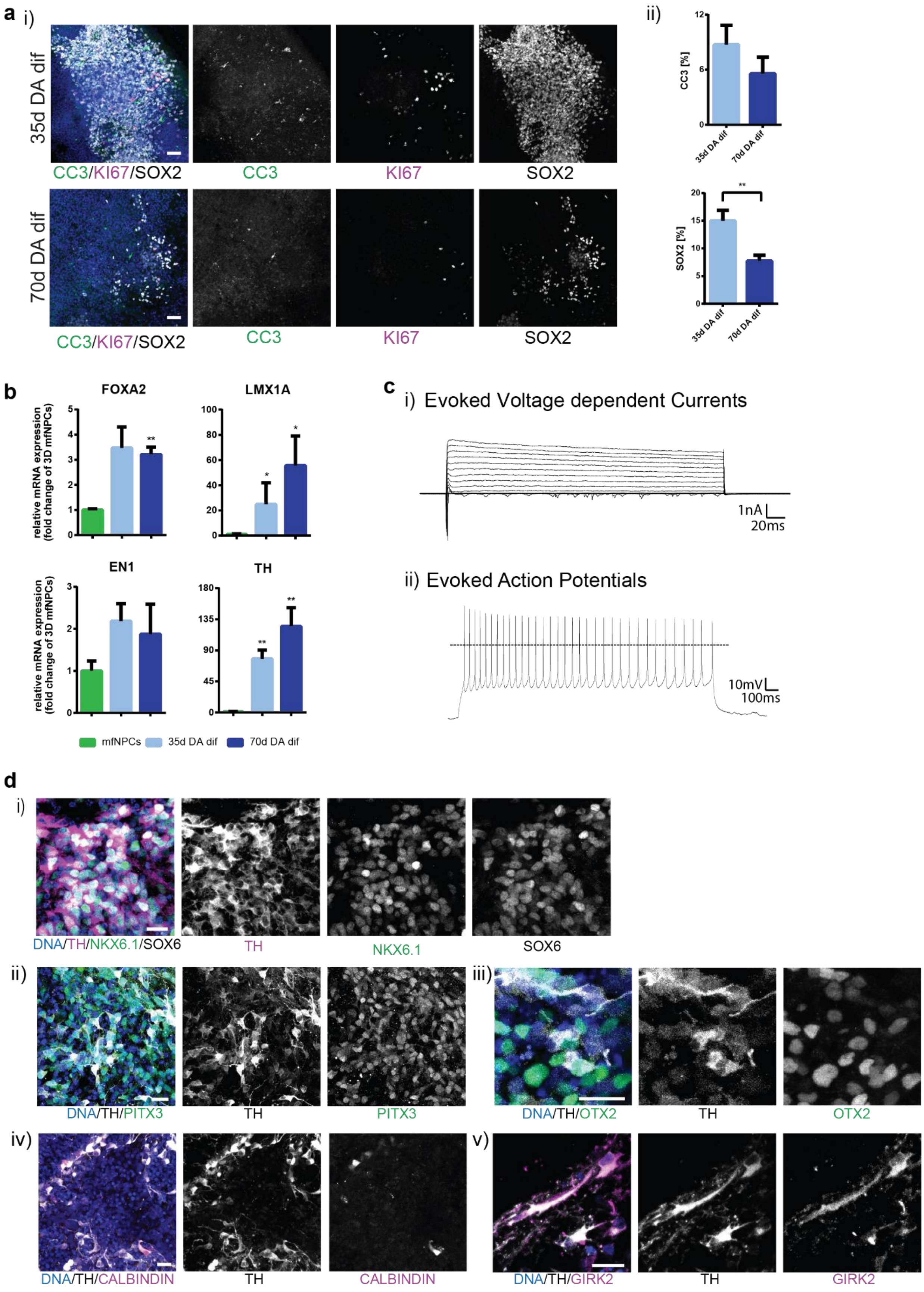

Supplementary Figure 3: Characterization of midbrain-specific organoids. Where indicated, analyses were performed with multiple cell lines (details see below) and a representative image looking similar to the images obtained from all used lines, is displayed. (a) (i) Immunohistological staining of apoptosis marker cleaved-caspase 3 (CC3), cell proliferation marker KI67, and stem cell marker SOX2 in an organoid quadrant. Scale bar is 50 µm. (ii) SOX2 and CC3 positive pixels expressed as a percentage of the total Hoechst signal (mfNPC lines H1-3, three passages each, n=9, here representative images are shown for line H1). (b) qRT-PCR analysis for mDAN markers FOXA2, LMX1A, EN1, and TH. Data presented as mean ±SEM, * p-value < 0.05, ** p-value < 0.01 (mfNPC lines H1-4, n=4). (c) Representative traces of voltage-gated sodium inward and potassium outward currents (i) evoked by stepwise depolarizations and sustained firing patterns of evoked action potentials (ii) upon a current-step application (i.e. 50 pA). These recordings were obtained from the same cell displayed in Figure 1 from a dissociated 3D culture of 75 days from line H4. Black plain line in (ii) indicates 0 mV. Experiment conducted with mfNPC line H4, P2-GC and P3-GC, in total 26 cells were recorded. (d) Immunostaining for indicated ventral midbrain markers of 35 old organoid middle (i)-(iv) and edge sections (v). Scale bars are 20 µm. hMOs derived from mfNPC lines H1-4, here representative images are shown for line H1 (i and iii) and line H2 (ii, iv, v).

Supplemetary Table 1

cell lines	Derivation conditions	Gender	Age at sampling	Genotype	Comment	hiPSC ID	Figure
H1	2D	Female	81	LRRK2 WT	Reinhardt *et al.*, 2013	2.0.0.10.1.0	S1C, D, S2B, C
	3D	Female	81	LRRK2 WT	Reinhardt *et al.*, 2013	2.0.0.10.1.0	1B, C, E, F, G, S3A, B, D
H2	3D	Male	n.a	LRRK2 WT	Alstem (iPS15)	2.0.0.33.0.0	1B, C, E, G, S3A, B, D
H3	3D	Female	n.a.	LRRK2 WT	Bill Skarnes, WTSI	2.0.0.19.0.0	1B, C, E, G, H, 2A, B, C, S3A, B, D
H3-G2019S (isogenic to H3)	3D	Female	n.a.	LRRK2 G2019S	Qing *et al.*, 2017	2.0.8.19.0.7	2B, C
H4	3D	Female	cord blood	LRRK2 WT	Gibco (A13777)	2.0.0.15.0.0	1D, F, 2A, B, C, S3B, C, D
H4-G2019S (isogenic to H4)	3D	Female	cord blood	LRRK2 G2019S	Qing *et al.*, 2017	2.0.8.15.0.7	2B, C
P1-GC	2D	Female	81	LRRK2 WT	Reinhardt *et al.*, 2013	2.1.2.11.2.0	S1B, C, D, S2B, C, D
P2	3D	Female	54	LRRK2 G2019S	Reinhardt *et al.*, 2013	2.1.1.46.0.0	2A, B, C
P2-GC (isogenic to P2)	2D	Female	54	LRRK2 WT	Reinhardt *et al.*, 2013	2.1.2.46.0.0	S1B, C, D, S2B, C, D
	3D	Female	54	LRRK2 WT	Reinhardt *et al.*, 2013	2.1.2.46.0.0	1D, S3C, 2B, C
P3	3D	Female	81	LRRK2 G2019S	Reinhardt *et al.*, 2013	2.1.1.11.3.0	2A, B, C
P3-GC (isogenic to P3)	3D	Female	81	LRRK2 WT	Reinhardt *et al.*, 2013	2.1.2.11.3.0	1D, S3C 2B, C

Supplementary Table 1 related to experimental procedures: Cell lines used in this study to generate 2D or 3D cultures. Human mfNPCs were derived from iPSCs that have been previously published [1,2]. Our data set includes iPSCs of healthy or diseased origin (H=healthy, P=PD patient with LRRK2-G2019S mutation). Additionally, cells were derived from isogenic controls, with either inserted LRRK2 mutation (H-G2019S) or gene corrected LRRK2 mutation (P-GC). The last column shows the contribution of each cell line to the data in the respective figure.

Supplementary Table 2

Antibody	Species	Source	Ref.-No.	Dilution
Calbindin	mouse	swant	300	1:500
CC3	rabbit	Cell Signaling Technology	9661	1:200
CORIN	rat	R&D systems	MAB2209	1:500
DAT	rat	Abcam	Ab5981	1:200
DDC	rabbit	Thermo Scientific	PA5-25450	1:25
Dopamine	rabbit	ImmuSmol	IS1005	1:500
EN1	goat	Santa Cruz	sc- 46101	1:300
FOXA2	mouse	Santa Cruz	sc-101060	1:250
GIRK2	goat	Abcam	Ab65096	1:200
KI67	mouse	BD Biosciences	550609	1:200
LMX1A	rabbit	Abcam	ab139726	1:200
LMX1A	rabbit	Sigma	HPA030088	1:1000
NKX6.1	mouse	DSHB	F55A10	1 mg/ml
OTX2	goat	R&D	AF1979	1:500
OTX2	goat	R&D	AF1979	1:500
PAX3	mouse	DSHB	Pax3	1 mg/ml
PAX6	rabbit	Covance	PRB-278P	1:300
PITX3	rabbit	Millipore	AB5722	1:200
SOX1	goat	R&D systems	AF3369	1:100
SOX2	goat	R&D systems	AF2018	1:200
SOX2	goat	Santa Cruz	Sc- 17320	1:400
SOX6	rabbit	Sigma	HPA001923	1:200
TH	chicken	Abcam	ab76442	1:1000
TH	rabbit	Abcam	ab112	1:1000
TH	rabbit	Santa Cruz	sc-14007	1:1000
TH	rabbit	Santa Cruz	14007	1:300
TH	mouse	Immunostar	22941	1:100
TH	rabbit	Millipore	657012	1:400
TUJ1	mouse	BioLegend	801201	1:600
TUJ1	rabbit	Covance	PRB-435P-0100	1:600
TUJ1	chicken	Millipore	AB9354	1:600
TUJ1	mouse	Covance	MMS-435P-100	1:1000

Supplementary Table 2 related to experimental procedures: Antibodies used in this study.

Supplementary Table 3

Feature	Description
Related to Figure 1:	**Generation of midbrain-specific organoids**
**Nuclei mask**	Count of nuclear positive pixels.
**CC3 mask**	Count of cleaved-caspase 3 positive pixels.
**CC3 percentage**	CC3 mask / Nuclei mask expressed as percentage.
**SOX2 mask**	Count of SOX2 positive pixels.
**SOX2 percentage**	SOX2 mask / Nuclei mask expressed as percentage.
Related to Figure 2:	**Disease modeling of PD patient-derived midbrain-specific organoids**
**Nuclei mask**	Count of nuclear positive pixels.
**Nuclei mask high**	Count of nuclear positive pixels higher than the threshold (4000) identifying pyknotic nuclei.
**TH mask**	Count of TH positive pixels.
**TH skeleton**	Count of TH skeleton pixel (red). Skeleton is calculated using a thinning function resulting in a simplified representation of the neuronal branching. The skeleton allows identifying nodes and links. 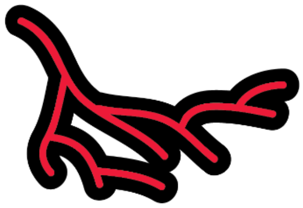
**Nodes**	Total number of points located at a bifurcation of neuronal branching (red points), generated from the TH skeleton. 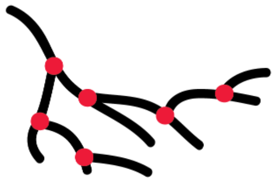
**Links**	Total number of connecting segments originated from the nodes (red lines), generated from the TH skeleton. 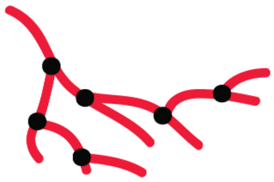
**TH+/Nuclei**	TH mask / Nuclei mask.
**TH percentage**	Percentage of number of neurons positive for TH. For this feature, nuclei

	were segmented with watershed function. Only nuclei segmented as single element were kept. The number of segmented nuclei positive for TH was calculated as a percentage of the total number of nuclei.
**TH Fragmentation**	Surface to volume ratio of TH mask.
**FOXA2 mask**	Count of FOXA2 positive pixels.
**FOXA2+/Nuclei**	FOXA2 mask / Nuclei mask.
**TH+/FOXA2+**	FOXA2 mask in TH positive neurons.
**TH+/FOXA2+/Nuclei**	FOXA2 mask identified in TH positive neurons/ Nuclei mask.
**TH-/FOXA2**	FOXA2 mask in TH negative perinuclear zone.
**TH-/FOXA2+/ Nuclei**	FOXA2 mask identified in TH negative neurons/ Nuclei mask.

Supplementary Table 3 related to Figure 1 and 2 and experimental procedures: Features from image analysis (adapted from Bolognin *et al.*, 2018[3]).

Supplementary Table 4

time point	comparison	THpos/FOXA2pos		Nodes		THneg/FOXA2pos	
d35	**H vs. H-G2019S**	ns	0.9851	****	< 0.0001	ns	0.8706
	**H vs. P-GC**	****	< 0.0001	****	< 0.0001	ns	0.1688
	**H vs. P**	****	< 0.0001	****	< 0.0001	****	< 0.0001
	**H-G2019S vs. P-GC**	****	< 0.0001	***	0.0007	ns	0.6202
	**H-G2019S vs. P**	****	< 0.0001	**	0.0022	***	0.0008
	**P-GC vs. P**	ns	0.9828	ns	0.9919	*	0.0239
d70	**H vs. H-G2019S**	ns	0.9995	ns	0.3920	ns	0.9544
	**H vs. P-GC**	**	0.0051	****	< 0.0001	ns	> 0.9999
	**H vs. P**	**	0.0041	****	< 0.0001	ns	0.6593
	**H-G2019S vs. P-GC**	**	0.0071	****	< 0.0001	ns	0.9627
	**H-G2019S vs. P**	**	0.0065	****	< 0.0001	ns	0.4017
	**P-GC vs. P**	ns	0.9940	ns	0.9986	ns	0.7679

Supplementary Table 4 related to Figure 2: Statistical evaluation of the image analysis for PD phenotyping. Several passages of PD patient-derived and healthy hMOs, as well as their isogenic controls (mfNPC H3, H3-G2019S, H4, H4-G2019S, P3, P3-GC, P4, and P4-GC, see Supplementary Table S1) were used. A 2way ANOVA, Tukey's multiple comparisons test was performed, asterisks and adjusted p values indicate significant differences between compared groups, * p-value < 0.05, ** p-value < 0.01, *** p-value < 0.001, **** p-value < 0.0001, ns= not significant.

Supplementary References

1 Qing, X., Walter, J., Jarazo, J., Arias-Fuenzalida, J., Hillje, A.-L. *et al.* CRISPR/Cas9 and piggyBac-mediated footprint-free LRRK2-G2019S knock-in reveals neuronal complexity phenotypes and α-Synuclein modulation in dopaminergic neurons. *Stem cell research* **24**, 44-50 (2017).

2 Reinhardt, P., Glatza, M., Hemmer, K., Tsytsyura, Y., Thiel, C. S. *et al.* Derivation and expansion using only small molecules of human neural progenitors for neurodegenerative disease modeling. *PloS one* **8**, e59252, doi:10.1371/journal.pone.0059252 (2013).

3 Bolognin, S., Fossepre, M., Qing, X., Jarazo, J., Scancar, J. *et al.* 3D Cultures of Parkinson's Disease-Specific Dopaminergic Neurons for High Content Phenotyping and Drug Testing. *Adv Sci (Weinh)* **6**, 1800927, doi:10.1002/advs.201800927 (2019).
